# Estrategia de control de la resistencia bacteriana a los antimicrobianos en Argentina

**DOI:** 10.26633/RPSP.2017.88

**Published:** 2017-06-19

**Authors:** Jaime Lazovski, Alejandra Corso, Fernando Pasteran, Mauricio Monsalvo, Julia Frenkel, Wanda Cornistein, Gonzalo Corral, Francisco Nacinovich

**Affiliations:** 1 Departamento de Salud Pública Universidad de Buenos Aires Buenos Aires Argentina Departamento de Salud Pública, Universidad de Buenos Aires, Argentina.; 2 Servicio Antimicrobianos, Laboratorio Nacional de Referencia en Resistencia a los Antimicrobianos Instituto Nacional de Enfermedades Infecciosas Argentina Servicio Antimicrobianos, Laboratorio Nacional de Referencia en Resistencia a los Antimicrobianos. Instituto Nacional de Enfermedades Infecciosas. Administración Nacional de Laboratorios e Institutos de Salud “Dr. Carlos G. Malbrán”, Argentina.; 3 Ministerio de Salud de la Nación Ministerio de Salud de la Nación Argentina Ministerio de Salud de la Nación, Argentina.; 4 Hospital Dr. Cosme Argerich Ciudad Autónoma de Buenos Aires Buenos Aires Argentina Hospital Dr. Cosme Argerich, Ciudad Autónoma de Buenos Aires, Argentina.; 5 Instituto Nacional de Epidemiología Instituto Nacional de Epidemiología Argentina Instituto Nacional de Epidemiología, Administración Nacional de Laboratorios e Institutos de Salud “Dr. Carlos G. Malbrán”, Argentina.; 6 Sociedad Argentina de Infectología Sociedad Argentina de Infectología Argentina Sociedad Argentina de Infectología, Argentina.

**Keywords:** Drug resistance, microbial, government programs, Argentina, Farmacorresistencia microbiana, programas de gobierno, Argentina, Resistência microbiana a medicamentos, programas governamentais, Argentina

## Abstract

*La aceleración observada en las últimas décadas sobre la emergencia y diseminación de la resistencia a los antimicrobianos está vinculada al abuso y/o mal uso de los antimicrobianos. En 2014, el Ministerio de Salud de Argentina, junto a otros organismos e instituciones, implementó una estrategia nacional para el control de la resistencia a los antimicrobianos con el objetivo de retrasar o impedir la emergencia y diseminación de bacterias resistentes. Este trabajo describe las acciones propuestas y los resultados obtenidos durante el primer período de implementación en materia de fortalecimiento de la vigilancia en salud humana, creación de una red de vigilancia en salud animal, planificación de la vigilancia del consumo de antimicrobianos, fiscalización de restricciones en la venta de estos, adecuación de las formas farmacéuticas a las necesidades de tratamiento, actualización del registro de antimicrobianos y de métodos de diagnóstico, restricción de su uso como promotores de crecimiento, promoción de su uso responsable, elaboración de guías de diagnóstico y tratamiento, creación de programas de gestión de antimicrobianos, y fortalecimiento de los programas de prevención y control de infecciones en establecimientos de salud y de producción agropecuaria*.

*Muchas de estas medidas son de implementación inmediata, particularmente en materia de regulación, fiscalización y gestión de antimicrobianos, y pueden reducir su uso innecesario y con ello el impacto sobre la resistencia a los antimicrobianos*.

## Abstract (es)

The accelerated emergence and spread of antimicrobial resistance observed in recent decades is associated with the abuse and/or misuse of antimicrobial drugs. In 2014, Argentina’s Ministry of Health, in conjunction with other agencies and institutions, rolled out a national antimicrobial resistance control strategy designed to slow or prevent the emergence and spread of resistant bacteria. This article describes the action proposed and results obtained during the first implementation period in terms of improving human health surveillance, creating an animal health surveillance network, planning antimicrobial drugs consumption surveillance, monitoring restrictions on sales, adapting dosage forms to treatment needs, updating the antimicrobial drugs registry and diagnostic methods, restricting the use of these drugs as growth promoters, encouraging responsible use, preparing diagnostic and treatment guidelines, creating antimicrobial drugs management programs, and strengthening infection prevention and control programs in health facilities and livestock production.

Many of these measures, particularly those related to antimicrobial drugs regulation, control, and management, can be implemented immediately, reducing the unnecessary use of these products, and with it, the impact on antimicrobial resistance.

## RESUMO

O aceleramento observado nas últimas décadas no surgimento e na disseminação da resistência aos antimicrobianos está vinculado ao uso excessivo e/ou mau uso dos antimicrobianos. Em 2014, o Ministério da Saúde da Argentina, junto com outros órgãos e instituições, implementou uma estratégia nacional para o controle da resistência aos antimicrobianos com o objetivo de retardar ou impedir o surgimento e disseminação de bactérias resistentes. Este estudo descreve as ações propostas e os resultados obtidos no primeiro período de implementação no que se refere ao reforço da vigilância em saúde humana, criação de uma rede de vigilância em saúde animal, planejamento da vigilância do uso de antimicrobianos, fiscalização das restrições na venda de antimicrobianos, adequação das formas farmacêuticas à necessidade de tratamento, atualização do registro de antimicrobianos e de métodos diagnósticos, restrição do uso de antimicrobianos como promotores de crescimento, incentivo ao uso responsável dos antimicrobianos, elaboração de guias de diagnóstico e tratamento, criação de programas de controle de antimicrobianos e fortalecimento dos programas de prevenção e controle de infecções em estabelecimentos de assistência à saúde e de atividade agropecuária.

Muitas das medidas são de implementação imediata, em particular a regulamentação, fiscalização e controle dos antimicrobianos, e podem reduzir o uso desnecessário e consequentemente o impacto na resistência aos antimicrobianos.

La gran aceleración observada en la última década en la emergencia y la diseminación de la resistencia a los antimicrobianos (RAM) tiene una vinculación directa con el abuso y el mal uso de estos agentes terapéuticos (1, 2). Se estima que 50% de todos los antimicrobianos (ATM) que se prescriben son innecesarios o se usan de manera inadecuada (3). Las causas de esto son, entre otras, la indicación de antibióticos en infecciones que no lo requieren, la presión que ejercen el paciente o sus familiares por insuficiente comprensión de la utilidad de los ATM, la falta de pruebas apropiadas de diagnóstico y el uso creciente de ATM con fines no terapéuticos en la producción intensiva de animales destinados al consumo humano (4–6).

Entre otras consecuencias, la RAM produce falla de tratamientos empíricos, incremento de la morbimortalidad y de los costos de atención, demora en la instauración de tratamientos adecuados, mayor uso de ATM de amplio espectro y alto costo, necesidad de indicar ATM con farmacocinética poco conocida y fracaso de procedimientos médicos que dependen de la efectividad de los ATM (por ejemplo, quimioterapia, trasplantes, diálisis renal, etc.) (7).

Los casos de RAM más difundidos, y los más graves en términos de morbilidad y mortalidad, se relacionan con las bacterias. Diversos mecanismos genéticos, bioquímicos y fisiológicos pueden ser responsables de RAM, pero el que genera mayor preocupación por su asociación a resistencia extrema o panresistencia es la producción de enzimas betalactamasas, en especial las carbapenemasas de los tipos *Klebsiella pneumoniae* (KPC), oxacilinasa (OXA) y metaloenzimas (Nueva Delhi metaloenzima, NDM) (8–10). La reciente detección del mecanismo de resistencia transferible a colistín (*mcr*1) en cepas de *Escherichia coli* aisladas en Argentina ha generado nuevas incertidumbres en cuanto a sus posibilidades terapéuticas (11). Las infecciones por bacterias resistentes están presentes en todos los países y no se limitan a los establecimientos de salud, por ejemplo, los casos de *Staphylococcus aureus* de la comunidad resistentes a la meticilina superan ahora en prevalencia a los hospitalarios (12). Por otra parte, diversos estudios demuestran que el ambiente es el principal reservorio de bacterias con resistencia adquirida y de genes que codifican mecanismos de resistencia (13–16).

**FIGURA 1.**
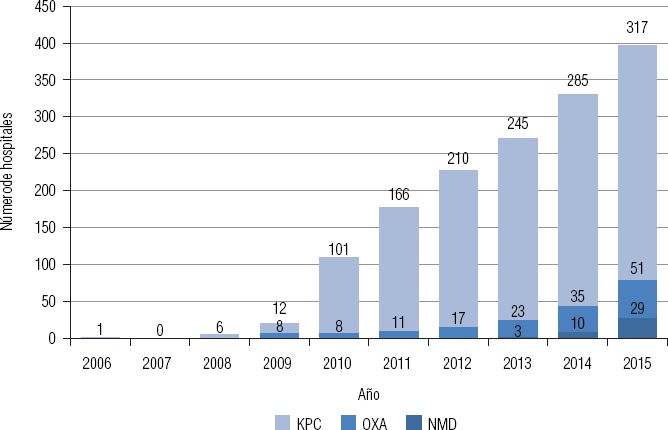
Número acumulativo de hospitales con aislamiento de enterobacterias productoras de carbapenemasas en Argentina, 2006–2015.

***Fuente:*** elaboración propia en base a datos del Laboratorio Nacional de Referencia.

KPC, Klebsiella pneumoniae carbapenemasa; OXA, carbapenemasa de naturaleza oxacilinasa; NDM, Nueva Delhi metaloenzima.

**FIGURA 2.**
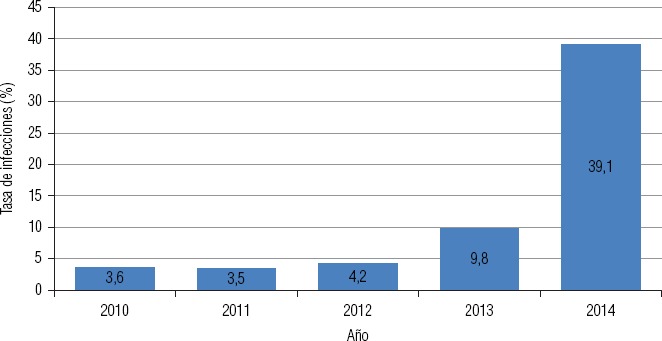
Evolución de la tasa de infecciones por bacterias productoras de carbapenemasas tipo KPC cada 10 000 egresos-año en hospitales de la ciudad de Buenos Aires, Argentina, 2010–14

***Fuente:*** elaboración propia en base a datos de la Red de Vigilancia de la Resistencia (WHONET, por sus siglas en inglés).

En años recientes, varios gobiernos y organismos internacionales han reconocido la gravedad del problema de la RAM y han elaborado planes para combatirlas; entre ellos se encuentra la iniciativa de un plan regional para las Américas que prepara la Organización Panamericana de Salud (OPS). Sin embargo, las acciones efectivas son aún insuficientes (17, 18).

A comienzos de 2014, el Ministerio de Salud de Argentina convocó a representantes de diferentes organismos, universidades y sociedades científicas para elaborar una estrategia de control de la RAM. El documento inicial fue sometido a consideración de otras entidades oficiales y comunitarias involucradas en el tema, y luego aprobado por resolución conjunta de los ministerios de Salud y de Agroindustria (19, 20). Mediante esta resolución se creó la Comisión Nacional para el Control de la RAM, cuya misión es vigilar el cumplimiento de la estrategia, además de proponer y actualizar las medidas de control. El propósito de este artículo es difundir la estrategia implementada y algunos de los resultados alcanzados.

## SITUACIÓN DE LA RAM EN ARGENTINA

El problema de la RAM es acuciante en Argentina. En la figura 1 se muestra el incremento del número de hospitales con casos o brotes de enterobacterias productoras de carbapenemasas de tipos KPC, OXA y NDM cuyo primer aislamiento fue confirmado por el Laboratorio Nacional de Referencia en Resistencia a los Antimicrobianos (LNR). En el caso de KPC, desde el primer aislamiento en 2006, el número de hospitales afectados ascendió a 317 en 2015. Cabe destacarse que este incremento no obedeció a mejoras en los procesos de detección (21).

En la figura 2 se observa que la tasa de infecciones causadas por bacterias productoras de KPC cada 10 000 egresos-año se ha incrementado diez veces en el período 2010–2014 en los hospitales de la Ciudad Autónoma de Buenos Aires.

## OBJETIVOS Y LÍNEAS ESTRATÉGICAS PARA EL CONTROL DE LA RAM

Los objetivos de la estrategia nacional son:

- Retrasar o impedir la emergencia y diseminación de bacterias resistentes mediante la regulación y fiscalización de la venta de ATM, la promoción de su uso responsable y la prevención y el control de infecciones en hospitales y establecimientos agropecuarios.
- Fortalecer la vigilancia de la RAM y del uso de ATM.
- Promover la innovación en ATM, promotores no antibióticos del crecimiento y pruebas diagnósticas para identificar bacterias resistentes.

Las líneas estratégicas y las acciones planteadas se describen a continuación y se resumen en el cuadro 1 bajo el esquema “Diez mandamientos para combatir la RAM”, tomando el concepto de los “Diez mandamientos para el uso adecuado de los antibióticos en la práctica médica ambulatoria” de la Sociedad Internacional de Quimioterapia (22).

### Vigilancia de la RAM

Argentina cuenta con una extensa red de laboratorios para la vigilancia de la RAM en salud humana, denominada WHONET-Argentina (en referencia al *software* del mismo nombre que se utiliza en esa actividad). La red funciona desde 1986 y está constituida por 95 laboratorios de los principales hospitales de Argentina. El Servicio Antimicrobianos del Instituto Nacional de Enfermedades Infecciosas (INEI) es el LNR y es la referencia regional para la Red de Vigilancia de la Resistencia a los Antimicrobianos en América Latina (RELAVRA), propiciada por la OPS. En el INEI existen otras dos redes que aportan información de resistencia bacteriana: el Sistema de redes de vigilancia de los agentes bacterianos responsables de neumonía y meningitis (SIREVA-Argentina) y el Programa de vigilancia de la sensibilidad antimicrobiana de gonococo (PROVSAG) (23).

En el caso de la salud animal y la producción agroalimentaria, existen dos tipos de abordaje:

- los estudios epidemiológicos sobre RAM desarrollados por investigadores universitarios y del Instituto Nacional de Tecnología Agropecuaria (INTA); y
- el Programa de Control de Residuos, Contaminantes e Higiene de Alimentos del Servicio Nacional de Sanidad y Calidad Agroalimentaria (SENASA), que realiza la detección de ATM y de contaminantes microbianos en los alimentos destinados al consumo humano.

En 2015, como parte de la estrategia, el SENASA creó el Programa Nacional de Vigilancia de RAM en Animales de Producción, con base en frigoríficos y establecimientos de producción intensiva de animales.

### Vigilancia del uso de antimicrobianos

**FIGURA 3.**
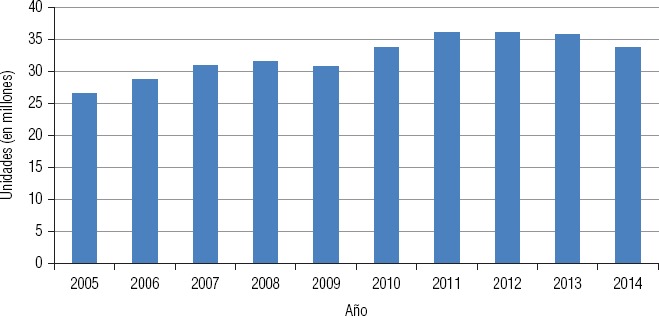
Evolución de las ventas de antimicrobianos en unidades farmacéuticas en Argentina, 2005–2014

En relación al uso de ATM, la información disponible sobre venta y consumo de ATM es fragmentada y tiene algunas limitaciones. Existen tres fuentes posibles:

- La consultora IMS Health, que recopila datos de comercialización de medicamentos en 13 500 farmacias de todo el país. En número de unidades, la venta de ATM muestra una tendencia creciente desde 2005, excepto para los años 2009, 2013 y 2014 (figura 3). Una limitación de estos datos es que no abarcan el subsector público, y solo reflejan el consumo ambulatorio. Los descensos detectados podrían deberse más a razones económicas (precio de los medicamentos, crisis económica) que a la aplicación de la presente estrategia, dado su carácter preliminar. Por otra parte, este descenso no impacta aún en la prevalencia de RAM, ya que para lograr ese efecto se requerirían plazos más largos.
- El programa Remediar del Ministerio de Salud, de alcance nacional, que provee una canasta básica de medicamentos a unos 7 900 centros de atención primaria de la salud de todo el país. Los datos de gestión permiten medir las tasas de uso de ATM por establecimiento y de prescripción por diagnóstico. La limitación de esta fuente es que abarca solo el subsector público y, dentro de este, exclusivamente a los centros de atención ambulatoria.
- El Instituto Nacional de Estadísticas y Censos (INDEC) del Ministerio de Hacienda, que releva las ventas de la industria farmacéutica en grandes grupos de medicamentos, aunque estos datos no discriminan las ventas al exterior.

**CUADRO 1.** “Diez mandamientos” para combatir la resistencia a los antimicrobianos

Mandamientos	Acciones
Asumir el liderazgo para la prevención y control de la RAM	Elaborar y conducir una estrategia para la prevención y control de la RAM con participación de expertos. Financiar las actividades de la estrategia.
Vigilar la RAM en salud humana y animal	Desarrollar una red nacional de vigilancia coordinada por laboratorios de referencia para salud humana, salud animal y producción agroalimentaria.
Vigilar el consumo de ATM	Desarrollar un sistema de vigilancia de ventas, acceso y utilización apropiada y adecuada de ATM.
Regular y fiscalizar la venta y utilización de ATM	Establecer exigencia de venta bajo receta y fiscalizar su cumplimiento efectivo. Regular sobre la cantidad de unidades en las formas farmacéuticas. Prohibir el uso de ATM como promotores de crecimiento en la producción agroalimentaria.
Promover el uso responsable de ATM	Elaborar guías de diagnóstico y tratamiento de infecciones prevalentes. Capacitar a los equipos de salud en el uso apropiado y adecuado de ATM y en las medidas de prevención y control de IACS.
Promover la participación comunitaria	Difundir la problemática en la población y educar sobre los usos precisos de ATM.
Implementar mecanismos de control del uso de ATM	Implementar programas de gestión de ATM en servicios de salud con y sin internación.
Fortalecer los programas de prevención y control de IACS	Desarrollar una estrategia de vigilancia de las IACS.Implementar programas de prevención y control de IACS en servicios de salud.
Promover la investigación de ATM y métodos de diagnóstico	Establecer la problemática de la RAM como prioridad para el financiamiento y la realización de investigaciones.Priorizar la evaluación de ATM y métodos diagnósticos.
Monitorear y evaluar el logro de los objetivos	Conformar una comisión que monitoree y evalúe la implementación y ejecución de la estrategia de control.

***Fuente:*** elaboración propia con base en Levy-Hara G et al (22).

ATM, antimicrobianos; RAM, resistencia a los antimicrobianos; IACS, infecciones asociadas a los cuidados de la salud.

***Fuente:*** elaboración propia con datos de IMS Health.

La estrategia nacional propone generar datos desde tres perspectivas: el consumo de ATM, el uso apropiado (indicación acorde al diagnóstico) y adecuado (utilización correcta de vías, dosis y duración) y los costos de atención. Para esto se utilizarán las fuentes disponibles de manera integrada, agregando estudios específicos que permitan conocer los usos apropiado y adecuado de ATM, y los costos de atención de las infecciones resistentes (24).

### Regulación y fiscalización de la venta de antimicrobianos

Entre los factores que inciden en el uso incorrecto de ATM se encuentran la automedicación, la dispensa sin prescripción y el incumplimiento de indicaciones médicas.

La Resolución 3835/1969 de la Secretaría de Salud Pública estableció que los medicamentos con actividad antibiótica de uso sistémico deben dispensarse bajo receta archivada por el expendedor, con penas al incumplimiento de: apercibimiento, multa, clausura, suspensión de la matrícula, inhabilitación y/o prisión de hasta tres años (Leyes 17565/1967 de Ejercicio de Farmacia y 26524/2009 del Código Penal). Debido a la organización federal de Argentina, las autoridades sanitarias de las 24 jurisdicciones del país son responsables de la fiscalización y aplicación de sanciones, aunque existe evidencia de un alto incumplimiento de estas normas (25).

Además de alentar a las autoridades jurisdiccionales al efectivo desempeño de sus funciones, se han emprendido acciones de sensibilización y capacitación dirigida a los farmacéuticos y las asociaciones profesionales y comerciales del sector.

Otro aspecto de la regulación se refiere a las formas farmacéuticas de dispensa. Es práctica habitual que el número de unidades contenidas en los productos no contemple la duración real de los tratamientos, favoreciendo de este modo el uso inadecuado de los ATM. Para controlar este efecto, la Administración Nacional de Medicamentos, Alimentos y Tecnología Médica (ANMAT) estableció en la Disposición 7.130/2015 que las presentaciones farmacéuticas de ATM deberán concordar con las dosis y duraciones de tratamiento usuales.

El proceso de registro de ATM y métodos de diagnóstico es otro de los aspectos a considerar. A causa de la RAM existe la necesidad de utilizar antiguos ATM por fuera de las indicaciones aprobadas o con escasa información de farmacocinética y farmacodinamia. En cuanto a los métodos diagnósticos, hubo retrasos en el proceso de aprobación, por ejemplo, de un método de detección rápida de bacterias resistentes con inhibidores de carbapenemasas (DCM-Brit®), desarrollado en Argentina por el LNR del INEI. Con el fin de subsanar estas falencias, ANMAT creó un grupo de trabajo conjunto con sociedades científicas para actualizar el registro de los ATM acorde las necesidades terapéuticas actuales y otorgarle prioridad a la evaluación y registro de métodos diagnósticos de la RAM.

Por último, acerca de la utilización de ATM en sanidad animal y producción agroalimentaria, el SENASA implementó recientemente diversas medidas destinadas a mejorar su control. Por ejemplo, la Resolución 609/2007 estableció que los ATM deben venderse solo bajo receta; la Resolución 666/2011 determinó que los establecimientos de producción de animales para consumo deben llevar un libro de registro de administración de productos veterinarios, sujeto a inspecciones; la Resolución 369/2013 creó el Sistema de Trazabilidad de Productos Fitosanitarios y Veterinarios, y la Resolución 594/2015 prohibió la inclusión de a ATM en los alimentos para animales a partir del 2 de enero de 2019. Esta última norma apunta a la futura prohibición total (en 2019) del uso de ATM como promotores de crecimiento animal.

### Promoción del uso responsable de antimicrobianos

El término “responsable” debe entenderse aquí como una extensión de la responsabilidad del profesional que prescribe (médico, veterinario, etc.) hacia todos los actores involucrados en el sector: farmacéuticos, microbiólogos, autoridades sanitarias, colegios y asociaciones de profesionales, industria farmacéutica, pacientes y la comunidad en general.

Desde su creación, la Sociedad Argentina de Infectología (SADI), junto con organizaciones gubernamentales y no gubernamentales, desarrolla múltiples actividades en el ámbito médico sobre el uso responsable de ATM. También lo hacen la Sociedad Argentina de Terapia Intensiva (SATI), la Sociedad Argentina de Pediatría (SAP) y la Asociación de Enfermeros en Control de Infecciones (ADECI).

Las líneas estratégicas que se proponen para el uso responsable de ATM son:

- Elaboración de guías de utilización de métodos de diagnóstico y tratamiento de infecciones destinadas a profesionales y equipos de salud.
- Promoción de la inclusión de la temática de la RAM en la currícula de las carreras de ciencias de la salud y otros ámbitos de capacitación profesional.
  El Ministerio de Salud nacional, la SADI y la SATI han realizado talleres de capacitación dirigidos a profesionales de salud en las principales ciudades argentinas, y un curso virtual gratuito con más de 2 500 participantes de distintas disciplinas afines y de diversos países de Latinoamérica. Además, se ha expuesto sobre RAM en congresos de diferentes especialidades médicas bajo la modalidad de mesas redondas o conferencias.
  En el caso específico del primer nivel de atención, el programa Remediar antes mencionado, realiza un curso de capacitación para equipos de salud sobre uso racional de la terapéutica en las infecciones ambulatorias. Un desafío a futuro será la inclusión de medidas de calidad que desalienten el uso de ATM en infecciones no bacterianas, pero para esto es necesario contar con pruebas de diagnóstico rápido, sencillas y económicas, que permitan definir la etiología de las infecciones más comunes en la práctica ambulatoria. Esto disminuiría la ansiedad de prescribir y recibir un ATM en ese ámbito (26).
- Programas de gestión de ATM en establecimientos de salud: estos han resultado muy útiles para reducir el uso innecesario o inadecuado de ATM, y además forman parte esencial de las medidas a promover. Lo ideal es que los programas de gestión de ATM actúen de modo coordinado con los comités de control de infecciones hospitalarias.
- Concientización comunitaria: implica la difusión de mensajes en medios masivos sobre la importancia de no consumir ATM sin consulta médica previa y de respetar las indicaciones de dosis y duración de los tratamientos. Un aliado importante para transmitir información clara y precisa es el periodismo especializado en salud. En 2015, el Ministerio de Salud convocó a representantes de diferentes organizaciones y a periodistas para elaborar consignas sobre los riesgos del uso excesivo de los ATM.
- Investigación: además de la necesidad de encontrar nuevas moléculas que reemplacen a las que van perdiendo efecto, hay otros aspectos de la RAM que requieren más información, por ejemplo, los estudios de farmacodinamia y farmacocinética de viejos ATM para optimizar su uso. En este marco, el Ministerio de Salud ha financiado ensayos terapéuticos en una variedad de infecciones para ofrecer alternativas racionales en estas situaciones y completar evidencia para la elaboración de guías y recomendaciones.

Respecto de la salud animal y producción agroalimentaria, el SENASA, las universidades, los colegios de veterinarios y los representantes de la Organización Internacional de Sanidad Animal (OIE) han tomado el compromiso de implementar acciones de capacitación, comunicación e investigación sobre el uso responsable de ATM en ese ámbito.

### Prevención y control de infecciones

Los programas de prevención y control de infecciones asociadas al cuidado de la salud (IACS) tienen más relevancia que nunca. Desde 1983, el Instituto Nacional de Epidemiología “Dr. Juan Jara” desarrolla el Programa Nacional de Epidemiología y Control de Infecciones Hospitalarias, con las siguientes actividades:

- Vigilancia continua de la IACS mediante el Programa de Vigilancia de Infecciones Hospitalarias de Argentina (VIDHA) en áreas críticas de 140 hospitales de todo el país, y la Encuesta anual de Prevalencia de Infecciones Hospitalarias de Argentina (ENPIHA) sobre áreas críticas y no críticas en los restantes establecimientos de salud.
- Implementación, fortalecimiento y asesoramiento a los comités de control y prevención de las IACS, promoviendo la extensión de sus funciones a la gestión de ATM para reducir su uso innecesario, en el marco de la calidad de atención y seguridad del paciente.

Bajo estos mismos preceptos, el SENASA y el INTA promueven el desarrollo de programas de prevención de infecciones en los establecimientos productivos.

## CONCLUSIONES

En pocas décadas, los ATM han pasado de ser “drogas milagrosas de gran impacto para la salud” a ser “un recurso no renovable y en vías de extinción”.

Los gobiernos deben renovar el compromiso con la salud pública a través de la ejecución de todos los mecanismos de vigilancia, regulación, fiscalización, capacitación, investigación y participación social a su alcance para prevenir y controlar el avance de la RAM. En Argentina se han activado estos mecanismos, que involucran a los distintos niveles de responsabilidad gubernamental, social, profesional y científica. En particular, las facultades de regulación y de convocatoria de la participación comunitaria muestran ya su efectividad en estas acciones.

En un escenario incierto y con perspectivas poco alentadoras, en el cual el efecto de la RAM amenaza con devolver a la humanidad a la era preantibiótica, sería promisorio que los países de la región de las Américas sumen sus esfuerzos en la lucha contra la RAM, aportando experiencia y creatividad, y potenciando la labor que cada uno desarrolla, habida cuenta de que en el mundo real no existen verdaderas fronteras para los problemas de salud.

Mientras tanto, la difusión de la problemática y las acciones emprendidas contribuirán a que la sociedad tome conciencia de los graves riesgos a los que se expone y empiece a utilizar los ATM de manera responsable.

## Agradecimientos.

Los autores desean agradecer a Gustavo Lopardo, Federico Luna, Silvia Boni, Gabriela Giacoboni, Martín Minassian, Luis Barcos, Florencia Pantozzi, Nora Mestorino, Elsa Mercado y Alejandro Soraci por su activa participación en el grupo de trabajo para la elaboración de la Estrategia Argentina para el Control de la Resistencia Antimicrobiana.

## Declaración.

Las opiniones expresadas en este manuscrito son responsabilidad del autor y no reflejan necesariamente los criterios ni la política de la *RPSP/PAJPH* y/o de la OPS.
